# Enhanced Binding of Poly(ADP-ribose)polymerase-1 and Ku80/70 to the *ITGA2* Promoter via an Extended Cytosine-Adenosine Repeat

**DOI:** 10.1371/journal.pone.0008743

**Published:** 2010-01-15

**Authors:** Yann Cheli, Shirley A. Williams, Robert Ballotti, Diane J. Nugent, Thomas J. Kunicki

**Affiliations:** 1 The Department of Molecular and Experimental Medicine, The Scripps Research Institute, La Jolla, California, United States of America; 2 Institut National de la Santé et de la Recherche Médical, Unité 895, Université de Nice, Nice, France; 3 Division of Hematology, The Children's Hospital of Orange County, Orange, California, United States of America; Baylor College of Medicine, United States of America

## Abstract

**Background:**

We have identified a cytosine-adenosine (CA) repeat length polymorphism in the 5′-regulatory region of the human integrin α2 gene *ITGA2* that begins at −605. Our objective was to establish the contribution of this polymorphism to the regulation of integrin α2β1 expression, which is known to vary several-fold among normal individuals, and to investigate the underlying mechanism(s).

**Methodology/Principal Findings:**

In combination with the SNP C-52T, previously identified by us as a binding site for the transcription factor *Sp1*, four *ITGA2* haplotypes can be distinguished, in the order in which they enhance *ITGA2* transcription: (CA)_12_/-52C>(CA)_11_/-52C>(CA)_11_/-52T>(CA)_10_/-52T. By DNA affinity chromatography and chromatin immunoprecipitation (ChIP) assays, we show that poly (ADP-ribose)polymerase-1 (PARP-1) and Ku80/70 bind specifically and with enhanced affinity to the longer (CA)_12_ repeat alleles.

**Conclusions/Significance:**

The increased binding of PARP-1 and Ku80/70, known components of transcription co-activator complexes, to the longer (CA)_12_ alleles of *ITGA2* coincides with enhanced α2β1 expression. The most likely explanation for these findings is that PARP-1 and Ku80/70 contribute to the transcriptional regulation of *ITGA2*. These observations provide new insight into the mechanisms(s) underlying haplotype-dependent variability in integrin α2β1 expression in human platelets and other cells.

## Introduction

The fact that cellular integrin α2β1 levels can vary up to ten-fold among normal, healthy subjects was first discovered on blood platelets [1], [2], where it leads to variation in adhesive function. Among patients with a genetic basis for impaired hemostasis or an increased propensity for thrombosis, differences in α2β1 can further influence risk for negative outcomes [3], [4], [5], [6]. However, the integrin α2β1 is expressed by a large variety of cell types, and comparable variation has now been observed in, for example, fibroblasts and keratinocytes, where it may have an impact on wound healing or other physiologic functions [7]. The genetic basis for this heritable variation has been the subject of a comprehensive study by our lab, and progress has been made in understanding the variety of mechanisms, some species-dependent [8], that control expression of this integrin.

We [1], [2] previously identified and characterized an *ITGA2* proximal promoter polymorphism at -52 (C-52T) that decreases significantly the binding of the transcription factor *Sp1*, known to be a key enhancer of *ITGA2* transcription [9]. A T at position -52 disrupts what is otherwise a highly favorable Sp1 binding site and decreases its binding by 8–10 fold [2]. This SNP, in linkage disequilibrium with two coding region SNPs, C807T [10], [11] and G1648A [12] defines five common and several rare *ITGA2* haplotypes [13].

The existence of variability in CA repeat length at this position in the *ITGA2* promoter was originally reported in abstract form by Sydor et al. [14], but not precisely defined. In the present report, we define the CA repeat length polymorphism as 10 to 12 repeats with the 3′ sequence beginning at −605, we show that it is in linkage disequilibrium with C-52T based on an analysis of 132 human chromosomes, and we analyze its contribution to transcription vis-à-vis C-52T in megakaryocytic (MK) and non-megakaryocytic (non-MK) cell lines.

## Results

### 5′-Regulatory Region CA Repeat Sequence

In this study, we have identified a polymorphic CA repeat sequence that begins at position −605 within the 5′-regulatory region of *ITGA2* (encompassing position 2878903 to 2878924 of NCBI NT_006713). Based on the sequence of 132 human chromosomes, there are three predominant alleles, and the frequency of each in a white, non-Hispanic population is: (CA)_10_ = 0.072; (CA)_11_ = 0.567; and (CA)_12_ = 0.361. A rare (CA)_13_ allele was detected only on two chromosomes. A comparison of these 132 human haplotypes confirmed complete linkage disequilibrium between -52C and (CA)_12_ and between -52T and (CA)_10_ (Chi-square = 80.016; p<0.001) (**Table 1**).

**Table 1:** Association of CA repeat length alleles with -52 C or -52T on 132 human chromosomes.

		CA Repeat Length		
		10	11	12
**-52**	**C**	0*	17	48
	**T**	16	51	0

* number of chromosomes positive for both alleles.

An obvious question is whether this modest variation in CA repeat length might have an influence on *ITGA2* transcription, independently of -52C/T (nucleotide 2879425 in NT_006713). To address this question, we cloned a 1.8 Kb segment of the *ITGA2* 5′-regulatory/promoter region (bp −1793 through +56) into the LUC reporter plasmid pgl2b.

**Figure 1:**
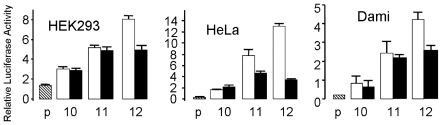
Transcriptional activity of *ITGA2* promoter-luciferase reporter constructs transfected into HEK293, HeLa, or Dami cells. A luciferase reporter assay was used to compare transcriptional activity in the presence of -52C (white bars) or -52T (black bars) within the *ITGA2* 5′-regulatory region (from −1793 to +56) containing (CA)_10_ (10), (CA)_11_ (11) or (CA)_12_ (12) repeat sequences (abscissa). The plasmid vector (p) lacking an insert served as a baseline (negative) control. Relative luciferase activity is indicated on the ordinate. The mean ± SD of three experiments is represented.

To analyze the combined effect of the CA repeat polymorphism and C-52T, six variants of the 1.8 Kb segment were created, each bearing 10, 11 or 12 CA repeats at the −605 site and either T or C at position -52. The relative activity of these constructs was measured in three human cell lines with very different backgrounds: HEK293, a human embryonic kidney epithelial cell line; HeLa, a cervical cancer cell line; and Dami, a human megakaryocytic cell line (**Figure 1**).

In the context of -52C (white bars), overall transcriptional activity was increased, as expected. In HEK293, mean luciferase activity in the presence of the (CA)_12_ repeat is 1.5- and 2.7-fold higher than that obtained in the presence of the (CA)_11_ and (CA)_10_ repeat, respectively. In Dami cells, the corresponding increases, 1.8- and 5.3-fold, are more dramatic. In HeLa cells, the equivalent increases are 1.6-fold and 8.1-fold.

In the context of -52T (black bars), overall transcriptional activity was significantly attenuated in all cell lines (**Figure 1**). The presence of the (CA)_12_ repeat still had an incremental effect on luciferase activity in Dami cells, with 1.2- fold and 4.3-fold increases relative to that observed with the (CA)_11_ or (CA)_10_ repeat, respectively. On the other hand, in either HEK293 or HeLa cells, there was no increase in luciferase activity between the (CA)_12_ repeat and the (CA)_11_ repeat, and only a modest increase in activity relative to the (CA)_10_ repeat (1.8- and 2.2-fold, respectively).

Taken together, these results indicate that the CA repeat sequence of itself has little effect on transcriptional activity under conditions where Sp1 binding would be minimized (in the presence of -52T). On the other hand, when Sp1-driven transcription is optimized by the presence of -52C, increasing CA repeat length enhances that activity.

### Association of CA Repeat Number with Platelet α2β1 Expression

**Figure 2:**
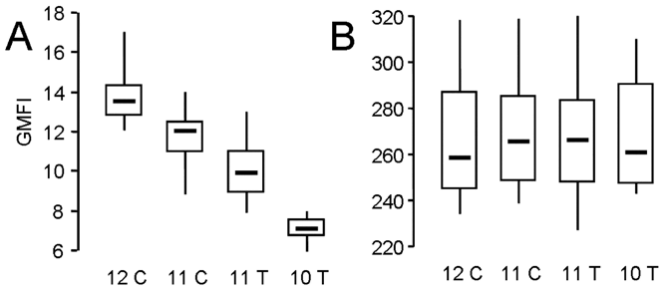
Platelet α2β1 surface expression measured by flow cytometry in whole blood. The geometric mean fluorescence intensity (GMFI) is plotted on the ordinate. ***A.*** Surface content of integrin α2β1 was measured by the binding of murine monoclonal antibody 12F1. ***B.*** Expression of integrin αIIbβ3 was measured by the binding of murine monoclonal antibody AP2. Blood was analyzed from donors who are homozygous for the following *ITGA2* haplotypes: (CA)_12_/-52C (12 C) (n = 21); (CA)_11_/-52C (11 C) (n = 14); (CA)_11_/-52T (11 T) (n = 27); and (CA)_10_/-52T (10 T) (n = 8). For each dataset in these box plots, the broad horizontal bar represents the median, the box represents the second and third quartiles, and the vertical line represents the range. In *A*, all pair wise comparisons are statistically significant (p<0.001); in *B*, all pair wise comparisons are not statistically significant (p>0.95). Comparable results were obtained with murine monoclonal antibodies 6F1 and 8C12, specific for integrin α2β1 (not shown).

We obtained the strongest confirmation of an effect of CA repeat length on *ITGA2* expression by measuring the level of platelet surface α2β1 (**Figure 2A**) in comparison to the level of platelet αIIbβ3 (**Figure 2B**) between normal subjects with known *ITGA2* haplotypes. To simplify the analysis, donors were selected who are homozygous for both CA repeat length and the C-52T allele; to minimize any effect of sample manipulation, platelet surface α2β1 content was measured in whole blood, as previously described [10].

The data are presented as box plots to present the maximum amount of statistical information in a visual format. Donors are grouped into four datasets, comparing donors homozygous for (CA)_12_ and -52C (n = 21), for (CA)_11_ and -52C (n = 14); for (CA)_11_ and -52T (n = 27); and for (CA)_10_ and -52T (n = 8). The descriptive statistics are summarized in **Table 2**. First, the levels of platelet integrin αIIbβ3 were virtually identical between groups (**Figure 2B**) (p>0.98). On the other hand, the level of integrin α2β1 measured with 12F1 declined progressively with decreasing CA repeat length and the presence of -52T (**Figure 2A**). The same findings were made with 6F1 and 8C12 (**Table 2**). All pair-wise comparisons for each monoclonal antibody are statistically significant (p<0.05). These results strongly support the notion that the CA repeat length polymorphism has an independent influence on the rate of *ITGA2* transcription.

**Table 2:** Surface content of platelet integrins α2β1 and αIIbβ3 measured in whole blood by flow cytometry.

			12F1		6F1		8C12		AP2	
CA	-52	n	Mean	SD	Mean	SD	Mean	SD	Mean	SD
**12**	**C**	21	13.7*	1.3	16.4	1.9	23.1	2.5	267.5	26.3
**11**	**C**	14	11.7	1.5	13.4	2.1	16.0	2.4	270.4	26.1
**11**	**T**	27	10.2	1.4	11.2	1.3	13.7	1.6	267.2	23.3
**10**	**T**	8	7.1	0.7	8.2	0.7	9.5	0.8	269.3	26.7

* Geometric Mean Fluorescence Intensity (GMFI).

The background GMFI obtained with isotype-matched non-immune mouse IgG has been subtracted from these values.

Integrin α2β1 specific antibodies are 12F1, 6F1 and 8C12. AP2 is specific for integrin αIIbβ3.

### Identification of Proteins That Bind the CA Repeat Sequence In Vitro

**Figure 3:**
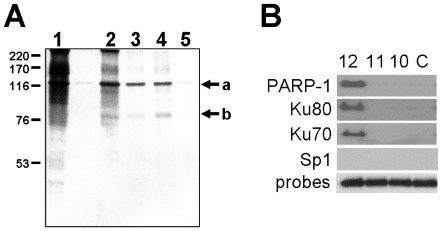
Identification of proteins that bind to the (CA)_12_ oligonucleotide sequence *in vitro*. ***A.*** One mg of Dami nuclear extract protein was incubated with one of four biotinylated (CA)_12_ oligonucleotide probes in binding buffer. Oligonucleotide/protein complexes were adsorbed to streptavidin-agarose beads, and the bound proteins were eluted, separated by SDS-PAGE and visualized using silver stain. The following protein samples are depicted: (lane1) Starting nuclear extract; (lane 2) Proteins bound to CA12 in the absence of calf thymus DNA; (lane 3) Proteins bound to CA12 in the presence of calf thymus DNA; (lane 4); Proteins bound to CA12 in the presence of calf thymus DNA+ 5-fold molar excess of control oligonucleotide; and (lane 5) Proteins bound to control oligonucleotide in the presence of calf thymus DNA. The two prominent protein bands with MWApp of 120 kDa and 80 kDa (positions indicated by arrows a and b, respectively, to the right of the panel) were excised and processed by MS/MS. Peptides recovered and sequenced by MS/MS are depicted in Figure S1. The positions held by molecular weight standards (Amersham Biosciences, Pittsburgh, PA) are indicated to the left of the panel. ***B.*** Dami nuclear extract proteins were incubated *in vitro* with the biotin-conjugated oligonucleotide probes: CA12, CA11, CA10 or control oligonucleotide (C). The resultant oligonucleotide/protein complexes were pulled down with streptavidin agarose. Nuclear proteins present in the complexes were separated by SDS-PAGE and identified by western blotting using the specific antibodies indicated to the left of the figure. The presence of PARP-1, Ku80 and Ku70 was confirmed in this manner. Antibodies specific for Sp1 served as a negative control, since the oligonucleotides used in these assays does not contain the Sp1 binding site. To confirm comparable oligonucleotide loading, the same samples were electrophoresed in a 2% agarose gel and stained with ethidium bromide (probes; negative image).

We used oligonucleotide affinity chromatography to capture proteins that bind to the (CA)_12_ repeat sequence *in vitro* (**Figure 3**). To eliminate non-specific binding, we performed the affinity chromatography in the presence of an excess of calf thymus DNA, as recommended by Kadonaga et al. [15]. Oligonucleotide/protein complexes were immobilized with streptavidin-Sepharose, and bound proteins were eluted from the oligonucleotide by addition of SDS and heating and then separated by SDS-PAGE (**Figure 3A**). The initial nuclear extract is depicted in lane 1. In the absence of calf thymus DNA, several proteins were complexed with biotin-CA12 (lane 2), including two prominent proteins with MWapp of 120 kDa (arrow a) and 80 kDa (arrow b). In the presence of calf thymus DNA, the same two proteins were still complexed with Biotin-CA12 (lane 3). The addition of a five-fold excess of control oligonucleotide together with Biotin-CA12 (lane 4) did not inhibit the binding of the 120 and 80 kDa proteins to Biotin-CA12. Finally, neither of these two proteins was complexed to the control oligonucleotide alone (lane 5).

MS/MS. The 120 and 80 kDa protein bands were individually extracted from the gel and subjected to tandem mass spectrometry (MS/MS). Based on electrophoretic mobility and the results of MS/MS, the two bands were identified as PARP-1 (120kDa) and Ku80 (80 kDa) (**Figure S1**).

The identities of the proteins present in purified biotin-CA12/protein complexes were confirmed by western blot, using antibodies specific for PARP-1, Ku80, Ku70, and Sp1 (**Figure 3B**). Ku70 (70 kDa), although not visible in the silver -stained gel in **Figure 3A**, is known to form a heterodimer with Ku80. Sp1, which is not present in any of the oligonucleotide/protein complexes, is not expected to bind to these CA repeat sequences and serves as a negative control. None of these proteins were detected in complexes formed with biotin-CA11, biotin-CA10 or the control oligonucleotide (**Figure 3B**).

### Chromatin Immunoprecipitation (ChIP)

**Figure 4:**
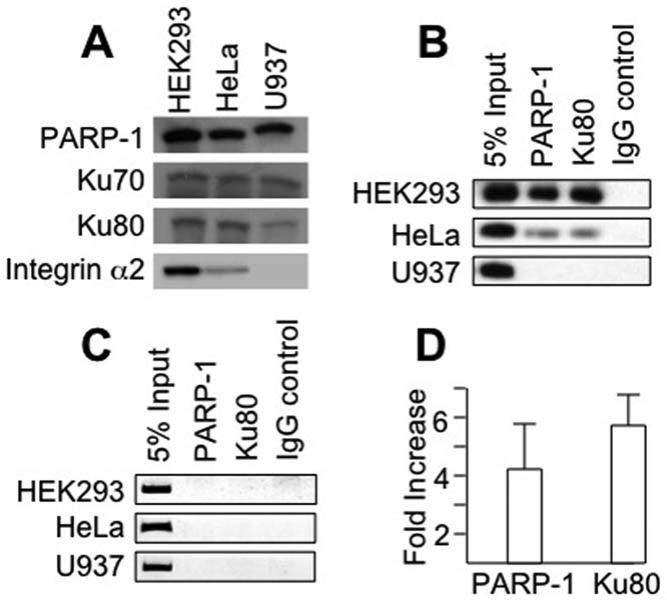
Chromatin Immunoprecipitation (ChIP). ***A.*** Confirmation of protein composition by Western Blot. The presence of (top row to bottom row) PARP-1, Ku70, Ku80, and integrin α2 in HEK293 cells, which express the *ITGA2* (CA)_12_ allele, HeLa cells, which express the *ITGA2* (CA)_11_ allele, or U937 cells, which do not express *ITGA2*, was confirmed by western blotting. The relative content of each protein in these three cell lines was comparable, except for the integrin α2, which is expressed at reduced levels in HeLa cells, and is absent in U937 cells. ***B.*** ChIP assays were performed using HEK293 cells (top row), HeLa cells (center row) and the control cell line U937 (bottom row). U937 bears the *ITGA2* (CA)_12_ allele, but does not express any detectable *ITGA2* mRNA, as determined by PCR (data not shown). Chromatin was sheared by sonication, and protein-DNA complexes were immunoprecipitated with antibodies against the PARP-1 or Ku80. The leftmost column represents 5% of total cross-linked chromatin before immunoprecipitation (5% input). Non-immune goat IgG served as a negative control (IgG control.). DNA retrieved after washing was amplified with primers specific for the test sequence encompassing the *ITGA2* CA repeat region (beginning at nucleotide −708 and ending at nucleotide −552). Data from one experiment representative of three independent experiments are depicted. ***C.*** ChIP assays were performed exactly as in ***B***, except that primers specific for a 3′-UTR negative control sequence were utilized. ***D.*** Semi-quantitation of PARP-1 and Ku80 bound to CA repeat sequences *in vivo*. The results of three ChIP assays such as that depicted in Figure 4B were analyzed semi-quantitatively. The relative binding of PARP-1 or Ku80 to the HEK 293 CA12 compared to HeLa CA11 sites is plotted on the ordinate as the fold-increase in density (HEK293/HeLa) of the photographic images corresponding to the amplified DNA sequences. Image densities were calculated using ImageJ software.

We used the ChIP assay to confirm that the co-activator protein complex is formed at the CA repeat sequence *in vivo* (**Figure 4**). We selected established cell lines that are homozygous for (CA)_12_ or (CA)_11_, but we have not yet identified a cell line that is homozygous for (CA)_10_. The HEK293 and U937 cell lines are both homozygous for the (CA)_12_ repeat, while HeLa cells are homozygous for the (CA)_11_ repeat. However, the U937 cell line serves as a negative control, because it is devoid of detectable α2β1 mRNA, even though it bears the *ITGA2* (CA)_12_ repeat. Previous results indicate that the U937 *ITGA2* promoter region is hypermethylated at CpG sites and transcriptionally silent [16].

By western blot assays (**Figure 4A**), comparable levels of PARP-1, Ku80 and Ku70 are detected in nuclear extracts of HEK293, HeLa or U937 cells. On the other hand, the level of integrin α2 is consistently higher in membrane extracts from HEK293 cells (roughly, 2-fold), relative to that found in HeLa cells, and absent from U937.

By ChIP, Ku80 and PARP-1 are specifically bound *in situ* to the (CA)_12_ site of HEK293 cells but associate much less strongly with the (CA)_11_ site of HeLa and do not associate with the (CA)_12_ of U937 (**Figure 4B**). In contrast, neither PARP-1 nor Ku80 bound to the negative control sequenced derived from the 3′-UTR (**Figure 4C**). As shown in **Figure 4D**, a semi-quantitative analysis based on three experiments indicates that there is, on average, a four-fold increase in bound PARP-1 and a six-fold increase in bound Ku80 at the HEK293 (CA)_12_ site relative to the HeLa (CA)_11_ site.

## Discussion

In this study, we have observed that an increase in the length of a CA repeat polymorphism in the 5′-regulatory region of *ITGA2* (beginning at −605) correlates with an increase in *ITGA2* transcription and a corresponding increase in the levels of platelet α2β1. This *ITGA2* CA repeat polymorphism and the downstream sequence -52C that enhances *Sp1* binding are in linkage disequilibrium.

The results of a luciferase reporter system clearly show that the length of the CA repeat correlates with the rate of transcription in synergy with the previously defined SNP at -52. The level of reporter activity increases when one compares the (CA)_11_ repeat to the (CA)_10_ sequence, but activity is most significantly enhanced in the presence of the (CA)_12_ repeat. Thus, four haplotypes can be distinguished, in the order in which they enhance *ITGA2* transcription: (CA)_12_/-52C>(CA)_11_/-52C>(CA)_11_/-52T>(CA)_10_/-52T.

*In vivo* confirmation of this relationship was obtained with blood platelets, in which the level of integrin α2β1 content correlates directly with the length of the CA repeat sequence in *ITGA2*. The combination of the CA repeat polymorphism and the -52 C/T SNP can readily account for the 3–4 fold variation in α2β1 expression that we have previously reported [1], [10] and confirm here.

Important information concerning the molecular basis for this effect was obtained by DNA affinity chromatography and chromatin immunoprecipitation (ChIP) analyses, whereby we show that PARP-1 and Ku80/70 bind specifically and most strongly to the (CA)_12_ repeat allele. The enhanced binding of PARP-1 and Ku80/70, known components of transcription co-activator complexes, with this allele of *ITGA2* suggests that both proteins are involved in the increased expression of α2β1 in platelets or other cells from donors who express the( CA)_12_ repeat allele. This novel finding provides new insight into an understanding of haplotype-dependent variability in integrin α2β1 expression.

PARP-1 binds to double-stranded DNA nicks, becomes activated, cleaves NAD+ into nicotinamide ADP-ribose, and polymerizes ADP-ribose on various nuclear proteins, including histones, certain transcription factors, such as Sp1, and itself [17]. This mechanism of poly ADP-ribosylation has been shown to contribute to various cellular processes, including DNA repair [18], transcriptional regulation [19], [20], [21], and cell cycle progression [21]. Ku80 and Ku70 form the heterodimeric DNA binding complex Ku80/70 that associates with PARP-1 and, among other functions, contributes to the regulation of gene transcription [22], [23], [24], [25], [26].

This study is the first to document that the presence of a CA repeat sequence that facilitates and/or enhances the specific binding of the PARP-1/Ku80/70 complex. Previously defined consensus Ku80/70 binding sites are GAGAAAGA [27], [28] or AAAAGGAAA and others [29]. These sequences are not present anywhere within 10 Kb of the 5′-regulatory region of *ITGA2*. Conversely, we are unaware of any previous report that CA repeats are a preferred binding sequence for either PARP-1 or Ku80/70.

Our study is not the first instance of a functional association between PARP-1 and integrin genes. Regulation of the expression of another integrin, αLβ2 (LFA-1; CD11a) by PARP-1 [30] may be implicated in the response to cellular damage by oxygen radicals or ischemia in neurons. Microglial migration is strongly controlled in living brain tissue by expression of this integrin, which is regulated by the formation of a nuclear PARP–1/NF-κB-protein complex. In addition, β1 integrin engagement by specific antibodies has been shown to enhance histone H3 acetylation in the mouse lung endothelial cell genome through a mechanism that requires PARP-1 [31].

## Materials and Methods

### Monoclonal Antibodies and Reagents

Murine monoclonal IgG antibody 6F1 (anti-α2β1) has been described [32] and is a gift from Dr. B. Coller (Rockefeller University, New York, NY). The murine hybridoma 12F1 producing IgG specific for α2β1 has been well characterized [33] and was generously provided by Dr V. Woods (University of California at San Diego, La Jolla, CA). The murine monoclonal IgG antibody 8C12, also specific for α2β1, was a gift from Dr. M. Ginsberg (University of California at San Diego, La Jolla, CA). AP2 is a murine monoclonal IgG antibody specific for the integrin αIIbβ3 developed and characterized in our laboratory [34]. Goat anti-human Ku80, goat anti-human PARP-1, rabbit anti-human Sp1, normal goat IgG and normal rabbit IgG were purchased from Santa Cruz Biotechnology (Santa Cruz, CA). The human megakaryocytic cell line Dami was obtained as previously described [2], and the human cell lines HeLa and HEK293 were obtained from ATCC (Manassas, VA).

### Measurement of Platelet Integrin Expression in Whole Blood by Flow Cytometry

Platelets were obtained from whole blood, as previously described [35], with prior informed consent and IRB approval. Murine monoclonal antibodies specific for the integrin α2β1 complex, 6F1, 12F1, and 8C12, were used to quantitate levels of this receptor on platelets by flow cytometry. Monoclonal antibody AP2 was employed to quantitate the integrin αIIbβ3. Each murine monoclonal antibody (0.5 µg in 10 µl PBS 7.4) was added to a 100 µl aliquot of whole blood anticoagulated with sodium citrate and let stand at ambient temperature for 60 minutes with constant, gentle mixing. FITC-F(ab′)_2_ goat anti-mouse IgG (heavy and light chains; Zymed) was then added (50 µl of a 1∶500 dilution), and the mixture let stand for an additional 30 minutes at ambient temperature with mixing. The mixtures were then diluted 1∶10 with PBS 7.4 and bound fluorescence was analyzed in a FACStar Plus (Beckman-Dickinson). Platelets were gated by forward versus side scatter, and the geometric mean fluorescence intensity (GMFI) of bound 12F1, 8C12, AP2, or nonimmune murine IgG was determined. The GMFI obtained for 12F1, 6F1, 8C12 or AP2 was corrected by subtracting from each the GMFI for nonimmune IgG.

The mean and standard deviation for GMFI values from each dataset were calculated. A Kruskal-Wallis one way analysis of variance (ANOVA) on ranks and a pairwise multiple comparison procedure (Holm-Sidak method) were used to determine the statistical significance of differences between means of each group.

### Plasmid Construction

A 1.9 Kb segment of the *ITGA2* 5′-regulatory/promoter region corresponding to nucleotides −1793 through +56 was amplified using genomic DNA as template and the primer pair:

Human A2.F = 5′-ATTAATGTGAGGCAG GAGTT-3′; and

Human A2.R = 5′-GAGGTTTGCAGAGGA TAC-3′.

Variants of this 5′-regulatory/promoter region construct were generated using the QuickChange II site-directed mutagenesis kit from Stratagene (La Jolla, CA) according to the manufacturer's instructions.

The polymerase chain reaction (PCR) was catalyzed by Platinum Taq high fidelity (Invitrogen, Carlsbad, CA). The amplicon was cloned into TOPO TA PCR2.1 vector (Invitrogen, Carlsbad, CA), according to the manufacturer's directions, and subcloned into the pGl2 basic vector (Promega, Madison, WI) at the *KpnI* and *XhoI* restriction sites.

### Cell Culture

Dami cells were grown in Iscove's modified medium supplemented with 10% (v/v) horse serum and 1% (v/v) Antibiotic-Antimycotic (Gibco-Invitrogen, Inc., Carlsbad, CA). HeLa and HEK293 cells were grown in Dulbecco's modified Eagle's Medium (DMEM) supplemented with 10% (v/v) Fetal Bovine Serum (FBS), 2 mM L-Glutamine and 1% (v/v) Antibiotic-Antimycotic (Gibco-Invitrogen, Inc.). In the case of HEK293 or HeLa, 5×10^4^ cells in 500 µl of medium were added to each well of a 24-well microtiter plate together with 200 ng of the pGl2 construct and 20 ng of the vector pRL-TK (Promega Corporation, Madison, WI). Transfection was initiated using Lipofectamine 2000 (Invitrogen, Carlsbad, CA), according to manufacturer's instructions. For Dami cells, 5×10^4^ cells in 350 µl of medium were added to one well of a 24-well plate together with 200 ng of the pGl2 construct and 20 ng of the pRL-TK vector. Transfection was initiated by addition of Effectene (Qiagen, Valencia CA), as previously described [36]. Dual luciferase assays (Promega) were performed according to the manufacturer's instructions. Luciferase activities were measured with the Clarity Luminescence Microplate Reader (Bio-Tek Instruments, Winooski, VE), and firefly luciferase activities were normalized to *Renilla* luciferase activities.

### Oligonucleotide Affinity Chromatography

All procedures were performed at 4°C. Nuclear extracts (1 mg total protein) in 1 ml of 25 mM Hepes, pH 7.8, containing 12.5 mM MgCl_2_, 2 mM dithiothreitol, 20% (vol/vol) Nonidet P-40 and 0.1 M KCl (Hepes buffer), as described by Kadonaga et al. [15], combined with sonicated calf thymus DNA (100 µg in 10 µl Hepes buffer), incubated for 10–15 minutes, and pre-cleared by adsorption with Streptavidin-agarose beads (Amersham, Piscataway, NJ) equilibrated in the same buffer. The agarose beads were pelleted by centrifugation, and the supernatants were combined with one of four double-stranded biotinylated-oligonucleotide probes in the same buffer for 1 hour on ice. The four oligonucleotide probes synthesized for this purpose were: the putative target sequence, Biotin-5′-TCTGT(CA)_12_GCT-3′ (CA12), Biotin-5′-TCTGT(CA)_11_GCTCT-3′ (CA11), Biotin-5′-TCTGT(CA)_10_GCTCTTG-3′ (CA10) and the negative control, Biotin-5′-TCTGT(CA)_5_GTGT(CA)_5_GCT-3′ (Control). The oligonucleotide/protein complexes were adsorbed to pre-blocked Streptavidin-agarose, and incubated for 30 min. The beads were then washed three times by successive incubation for 10 min in 2 ml buffer and centrifugation. After the final wash, the beads were pelleted, warmed to ambient temperature and resuspended in electrophoresis buffer containing 1% sodium dodecyl sulfate (SDS). Bound proteins were eluted by heating the samples for 30 min at 60°C. Eluted proteins were separated by SDS- polyacrylamide gel electrophoresis (SDS-PAGE), as previously described [10], and visualized using the SilverXpress staining kit (Invitrogen, Carlsbad, CA), according to the manufacturer's instructions. Protein identification was confirmed by subsequent western blot assays.

### Western Blot

Proteins in polyacrylamide slab gels separated by SDS-PAGE were transferred electrophoretically to a nitrocellulose membrane [10]. Membranes were blocked, immersed in a solution containing the primary antibody for 2 hours at room temperature, washed with gentle agitation, incubated in a solution containing the secondary antibody (diluted 1/5000) for 45 minutes at room temperature, and washed. Bound antibody was visualized by chemiluminescence.

### Mass Spectrometry

DNA affinity chromatography was performed with oligonucleotide CA12. Proteins were stained with the bio-safe-Coomassie blue staining kit (Bio-Rad, Hercules, CA), according to the manufacturer's instructions, and the two prominent bands with apparent molecular weights (MWapp) of 120 and 80 kilodaltons (kDa) were excised for identification.

Tryptic peptide mixtures were analyzed by microcapillary reverse phase chromatography coupled to an LCQ Deca XP MAX ion-trap mass spectrometer (ThermoFinnigan, San Jose, CA) using dynamic exclusion with MS/MS. Mass spectrometer was fully automated during the entire procedure using the Xcalibur 1.4 software system (ThermoFinnigan). Peptide identification was established using Bioworks browser version 3.1 (ThermoFinnigan) based on human databases.

### Chromatin Immunoprecipitation (ChIP) Assay

The details of ChIP assays are provided in the Supplemental materials (File S1).

## Supporting Information

Figure S1 Identification of proteins that bind to the (CA)12 oligonucleotide sequence in vitro by mass spectrometry. One hundred µg of Dami nuclear extract protein were incubated with the biotinylated (CA)12 oligonucleotide probe in binding buffer. DNA-protein complexes were adsorbed (pulled down) with streptavidin agarose beads, and the bound proteins were eluted, separated by SDS-PAGE and visualized using silver stain. Two higher MWApp protein bands (Band 1 and Band 2) were excised and processed by MS/MS. The Peptides recovered and sequenced by MS/MS are depicted. For each peptide sequence, the minimal cross correlation score (XC) and charge (Z) are displayed. XC is the confidence for identification according to the charge for the peptide recovered. Peptides in the upper list recovered from Band 1 identify it as PARP-1. Peptides in the lower list recovered from Band 2 and identify it as Ku80.
(0.41 MB TIF)

File S1 Supplemental Materials
(0.04 MB DOC)
