# Supplementary material for: Enhanced Binding of Poly(ADP-ribose)polymerase-1 and Ku80/70 to the ITGA2 Promoter via an Extended Cytosine-Adenosine Repeat
Source: PLoS One. 2010 Jan 15;5(1):e8743. doi: 10.1371/journal.pone.0008743 (PMC2806922; doi:10.1371/journal.pone.0008743)
Supplement: File S1 — Supplemental Materials (0.04 MB DOC) [file pone.0008743.s002.doc]

**SUPPLEMENTAL MATERIALS**

**Experimental Procedures**

*Chromatin Immunoprecipitation (ChIP) Assay-* Cells (3 x 10^7^) were washed once in 2 mM NaH_2_PO_4_ , 7.4 mM Na_2_HPO_4_, 138 mM NaCl and 2.7 mM KCl, pH 7.4 PBS), and resuspended in the same buffer. *In situ* cross-linking was accomplished by addition of 1% (w/v) (final concentration) p-formaldehyde (Sigma-Aldrich, St. Louis, MO) and incubation for 10 min at 37°C. The reaction was stopped by addition of glycine to a final concentration of 125 mM. Cells were washed in cold PBS and lysed in 1ml of 5 mM Hepes, pH 8, containing 85 mM KCl, 0.5% (w/v) Igepal-CA630 and 1 X complete protease inhibitor mixture (Roche Applied Sciences, Indianapolis, IN) (lysis buffer). Nuclei were pelleted by centrifugation at 3,500 x g for 5 min and resuspended to 1 x 10^7^ cells / ml in 50 mM Tris-HCl, pH8, containing 1% (w/v) sodium dodecyl sulfate (SDS), 10 mM ethylenediamine tetraacetic acid (EDTA), and 1 X protease inhibitor mixture (nuclei lysis buffer).

DNA was sheared by sonication to a size ranging from 0.2 Kb to 0.4 Kb. The sheared DNA solution was diluted 5-fold in 16.7 mM Tris-HCl, pH 8, containing 1.1% (v/v) Triton X-100, 1.2 mM EDTA, 167 mM NaCl, 0.01% (w/v) SDS, and 1X protease inhibitor mixture (immuno-precipitation buffer). One ml of the sheared DNA suspension (from approximately 2 x 10^6^ cells) was first incubated for 2 hours at 4^o^C with 80 μl of a 1:1 slurry of protein G agarose beads (Upstate, Temecula CA) in 10 mM Tris-HCl, pH 8, containing 1 mM EDTA plus 500 μg/ml of bovine serum albumin (BSA) and 100 μg/ml of nonimmune goat or rabbit IgG (Santa Cruz Biotechnology, Santa Cruz, CA). The mixture was centrifuged at 14,000 x g for 2 min, the supernatant was aspirated and saved, and the beads were discarded. Four μg of each polyclonal antibody was added to the supernatant, and these were incubated overnight at 4°C.

To adsorb the resulting immune complexes, the supernatants were then mixed with a fresh aliquot (60 μl of 1:1 slurry) of protein G agarose beads and incubated for 1 hour at 4°C. The beads were then pelleted by centrifugation at 9000 x g for 2 min at 4°C. The supernatant was removed, and the beads were rinsed by six successive centrifugation (each at 9000 x g for 2 min at 4°C) and resuspension steps (each for 5 min at 4°C under gentle agitation) in the presence of the following buffers: a) 20 mM Tris-HCl, pH 8, plus 1% (v/v) Triton X-100, 2 mM EDTA, 150 mM NaCl, and 0.1% (w/v) SDS (low salt washing buffer); b) 20 mM Tris-HCl, pH 8, plus 1% (v/v) Triton X-100, 2 mM EDTA, 500 mM NaCl, and 0.1% (w/v) SDS (high salt washing buffer ); c) 10 mM Tris-HCl, pH 8, plus 250 mM LiCl, 1% (v/v) Igepal ca-630, 1% (w/v) sodium deoxycholate, and 1 mM EDTA (low LiCl wash buffer); d) 10 mM Tris-HCl, pH 8, plus 500 mM LiCl, 1% (v/v) Igepal ca-630, (w/v) 1% sodium deoxycholate, and 1 mM EDTA (high LiCl wash buffer); and twice in e) 10 mM Tris-HCl, pH8, plus 1 mM EDTA (TE buffer).

Complexes of DNA and protein were eluted from the final bead pellet by incubation in 150 μl of 100 mM NaHCO_3_, 1% (w/v) SDS at 65°C for 15 min. Crosslinking was then reversed by addition of 12 μl of 5000 mM NaCl and incubation for 4 h at 65°C. The solubilized proteins were then digested, by addition of 90 μg/ml (final concentration) of proteinase K (Qiagen, Valencia CA) and incubation of the mixture for 1 h at 55°C. DNA was extracted from the mixture by addition of 1 volume of phenol: chloroform (1:1), pH 8, and precipitated by subsequent addition of 7.5 μg of glycogen (Ambion, Austin, TX), 0.5 volume of 3 M sodium acetate, and 2.5 volume of ethanol, and incubation for 1 hour at -80°C. The pelleted DNA was washed with 70% ethanol (v/v) and resuspended in 25 μl of water. The human *ITGA2* CA repeat region or the Sp1 binding site region were amplified by PCR using Taq polymerase (Qiagen, Valencia CA), according to the manufacturer's instructions. DNA in 5 μl samples was amplified using primers encompassing one of two sequences:

As the test sequence, the 5’ regulatory/promoter sequence from -708 to -552, using

CHIPA2F 5’-CTCCTAAACTCAGTCAT-3’ and

CHIPA2R 5’-CACAAGTTATGATTATCC -3’;

As a negative control sequence, a 3’-UTR segment from 106,328 to 106,461, using

CONTA2F 5’-CACCTTATTTTCTCTGTTCCTG-3’ and

CONTA2R 5’-CCATTTTATAATGAGGACTC-3’.

The products were separated by electrophoresis on a 3 % (w/v) agarose gel in 40 mM Tris, 20 mM acetic acid, 1 mM EDTA (TAE) and visualized by addition of 1 μg/ml ethidium bromide (Sigma). Images were analyzed using Gel doc XR and Quantity one 1-D analysis software (Bio-Rad, Hercules, CA). Quantification of PCR amplicons in photographic films derived from ChIP assays was performed by optical densitometry using the public domain software ImageJ , according to the developer’s instructions (http://rsb.info.nih.gov/ij/).

*Mass spectrometry-* For mass spectrometric protein identification, the DNA pull down procedure was performed with (CA)_12_ and scaled up 5-fold. Proteins were stained with the bio-safe-Coomassie blue staining kit (Bio-Rad, Hercules, CA), according to the manufacturer's instructions, and two prominent bands were selected and excised for identification.

Gel segments containing the selected proteins were diced into small pieces and destained by incubation in 100 μl of 25 mM NH_4_CO_3_ in 50 % (v/v) acetonitrile for 10 min. The procedure was repeated until the gel segments were free of color by visual inspection. Gels pieces were dried in a Vacufuge (Eppendorf, Westbury, NY) at 240 x g for 10min, and then incubated in 25 μl of 10 mM DTT in 25 mM NH_4_CO_3_ for 1 hour at 56°C to reduce the proteins. The supernatant was then aspirated and discarded, and the proteins in the gel slices were alkylated by addition of 25 μl of 55 mM iodoacetamide and incubation in the dark for 45 min at room temperature. The supernatant was aspirated and discarded, and the gel pieces were rinsed by addition of 100 μl of 25 mM NH_4_CO_3_ and incubation for 10 min. The supernatant was aspirated and discarded, and the gel pieces were dehydrated incubation in 100 μl of 25 mM NH_4_CO_3_ in 50 % (v/v) acetonitrile followed by centrifugation in the Vacufuge (Eppendorf, Westbury, NY) for 10 min at 240 x g. The dehydration step was then repeated.

Trypsin (Promega, Madison, WI) (0.5 μg in 5 μl of distillated water) was added to the dried gel pieces, which were then incubated for 10 min at 4°C. The gel pieces were then immersed in 25 mM of NH_4_CO_3_ and incubated for 18 hr at 37°C. Following this digestion, the supernatant was aspirated and collected into a clean polypropylene tube. The tryptic peptides were extracted by addition of 30 μl of 5% (v/v) acetonitrile, 0.5% (v/v) formic acid to the gel pieces, incubation for 30 min at 4^o^C (on wet ice), followed by sonication in a 22 ^o^C water bath for 5 min. The final sonication step was performed at least twice, and supernatants from each sonication step were combined.

Tryptic peptide mixtures were analyzed by microcapillary reverse phase chromatography coupled to an LCQ Deca XP MAX ion-trap mass spectrometer (ThermoFinnigan, San Jose, CA) using dynamic exclusion with MS/MS. Mass spectrometer was fully automated during the entire procedure using the Xcalibur 1.4 software system (ThermoFinnigan). Peptide identification was established using Bioworks browser version 3.1 (ThermoFinnigan) based on human databases. Spectral matches were retained with a minimal cross correlation score (Xcorr) of +/- 1.5, 2.00 and 2.5 for charges states (Z) +1, +2 and +3, respectively (Figure S1).
